# The Cause of so Many Failures

**Published:** 1874-12

**Authors:** 


*The Cause of so Many Failures.*—Dr. Black, speaking on this subject, says:

"There is one cause of failure to which men practicing in in country-towns are most subject, and that is that they are often undertaking operations which they can not get the time to properly complete. In bi-cuspids or molars, with pulps dead and roots filled, decayed deep in proximal surfaces, the retaining pits should be well in from the margin; if too near the margin it is almost impossible to avoid failure; should start at the retaining pits and gradually approach the margin, building up a thick sheet of gold so that it may not curl up and leave the margin under the blows of the mallet—causing failure."

---

### OBITUARY.

